# Association between Vitamin D supplementation and mortality in critically ill patients: A systematic review and meta-analysis of randomized clinical trials

**DOI:** 10.1371/journal.pone.0243768

**Published:** 2020-12-14

**Authors:** Liyuan Peng, Linjie Li, Peng Wang, Weelic Chong, Yin Li, Xi Zha, Haidong Deng, Huaqian Fan, Yu Zhang

**Affiliations:** 1 Affiliated Hospital of Chengdu University, Chengdu, Sichuan, China; 2 West China Hospital, Sichuan University, Chengdu, Sichuan, China; 3 Thomas Jefferson University, Philadelphia, Pennsylvania, United States of America; University of Florida, UNITED STATES

## Abstract

**Background:**

Observational studies suggest that low 25-hydroxyvitamin D status is common and has been associated with higher mortality in critically ill patients. This study aim to investigate whether vitamin D supplementation is associated with lower mortality in critically ill patients.

**Method:**

We searched Medline, Embase, and Cochrane databases from inception to January 12, 2020, without language restrictions, for randomized controlled trials comparing the effect of vitamin D supplementation with placebo in critically ill patients. Two authors independently performed data extraction and assessed study quality. The primary outcome was all-cause mortality at the longest follow-up.

**Result:**

We identified nine trials with a total of 2066 patients. Vitamin D supplementation was not associated with reduced all-cause mortality at the longest follow-up (RR 0.90, 95% CI 0.74 to 1.09, I^2^ = 20%), at 30 days (RR 0.81, 95% CI 0.56 to 1.15), at 90 days (RR 1.15, 95% CI 0.92 to 1.44), and at 180 days (RR 0.82, 95% CI 0.65 to 1.03). Results were similar in the sensitivity analysis. The sample size met the optimum size in trial sequential analysis. Similarly, supplemental vitamin D was not associated with length of ICU stay, hospital stay, or mechanical ventilation.

**Conclusion:**

Vitamin D supplement was not associated with reduced all-cause mortality in critically ill patients.

**Systematic review registration:**

Open Science Framework https://osf.io/bgsjq

## Background

Vitamin D plays an important role in maintaining the normal function of neurologic, cardiovascular, respiratory, and immune system [1, 2]. Observational studies have indicated that vitamin D deficiency is common in critically ill patients and is associated with mortality and length of ICU stay [3, 4]. Early randomized clinical trials (RCTs) individual showed lower observed mortality than placebo, although the differences were not significant [5–10]. In 2017 and 2018, three systematic reviews [11–13] have discussed the association between vitamin D supplementation and the most important clinical outcomes: all-cause mortality. One review [12] has shown benefit of vitamin D on survival, while two reviews [11, 13] have not shown the benefit. The ongoing debate has been fueled by the recent publications, two large RCTs [5, 14]. Thus, we performed a systematic review and meta-analysis to assess the effect of vitamin D compared to placebo on mortality.

## Method

### Protocol and guidance

This systematic review and meta-analysis has been reported in accordance with the preferred reporting items for systematic reviews and meta-analyses (PRISMA) statement (eTable 1 in S1 Appendix) [15]. We registered a protocol for the review in Open Science Framework https://osf.io/bgsjq.

### Eligibility criteria

Eligible studies met the following PICOS (participants, interventions, comparators, outcomes, and study design) criteria: (1) Population: adults admitted to the intensive care unit. (2) Intervention: administration of vitamin D, without restrictions in the type, dose, duration, or route of administration. (3) Comparison: placebo or no treatment. (4) Outcome: The primary outcome was all-cause mortality at the longest follow-up; second outcomes included mortality at 30 days, mortality at 90 days, mortality at 180 days, ICU mortality, in-hospital mortality, length of hospital stay, length of ICU stay, and length of mechanical ventilation. (5) Study design: RCT.

### Search strategy

We did computerized literature searches of Medline, Embase, and Cochrane CENTRAL Register of Controlled Trials from inception through January 12, 2020, without any language restrictions. The reference lists of included studies and reviews were searched for additional studies. Finally, we searched the World Health Organization’s International Clinical Trials Registry Platform to identify ongoing trials and evaluate the possibility of publication bias. The details of the search strategy are in eTable 2 in S1 Appendix.

### Study selection

After removal of duplicates, two investigators (LP and PW) screen each title and abstract independently and in duplicate. The full texts of the remaining studies were also assessed independently and in duplicate by the two authors (LP and PW). Discrepancies were resolved through discussion among the study team.

### Data extraction

Two investigators (HD and HF) independently and in duplicate extracted data about study characteristics, outcomes, and funding sources from the eligible trials using a predesigned spreadsheet for further analysis. Discrepancies were resolved through discussion among the study team. Correspondent authors of trials were contacted for unclear information and additional information that did not report outcomes of interest.

### Risk of bias

Risk of bias assessment was conducted by two investigators (LP and PW) using Cochrane Collaboration risk of bias tool 2 across seven domains. (https://methods.cochrane.org/bias/news/rob-2-tool) The individual domains included (1) random-sequence generation, (2) allocation sequence concealment, (3) blinding of participants and personnel, (4) blinding of outcome assessment, (5) completeness of outcome data, (6) selective reporting, and (7) other sources of bias.

### Data synthesis

All meta-analyses were conducted using Review Manager version 5.4 (Cochrane Collaboration). We calculated the relative risk (RR) with 95% CI for dichotomous outcomes and the mean difference with 95% CI for continuous outcomes. I^2^ values were calculated to estimate variation among studies attributable to heterogeneity. We calculated pooled effect sizes using random-effects models regardless of the value of I^2^ in a meta-analysis. We planned to examine publication bias via funnel plots (visually) and more formally with the Begg test and Egger test.

### Subgroup analysis

We conducted some subgroup analyses to test interactions according to the dose (≥300000IU or <300000IU), baseline 25 hydroxyvitamin D (≥20 or <20 ng/ml) and the route of administration (oral, intravenous, or intramuscular).

### Sensitivity analyses

We conducted sensitivity analyses to test the robustness of the findings included the following: using fixed-effect models, using absolute risk, and excluding trials at each time.

### Trial sequential analysis

We conducted a trial sequential analysis for the primary outcome. An optimal information size set to an overall 5% risk of type I error, 80% power, and relative risk reduction of 20%. Trial sequential analysis was done using Trial Sequential Analysis v.0.9.5.10 beta (Copenhagen Trial Unit, Copenhagen, Denmark).

### Quality of evidence

The grading of recommendations assessment, development and evaluation (GRADE) methodology was used for assessing the quality of evidence by two investigators (LP and PW) [16].

## Result

### Study selection and study characteristics

**Fig 1:**
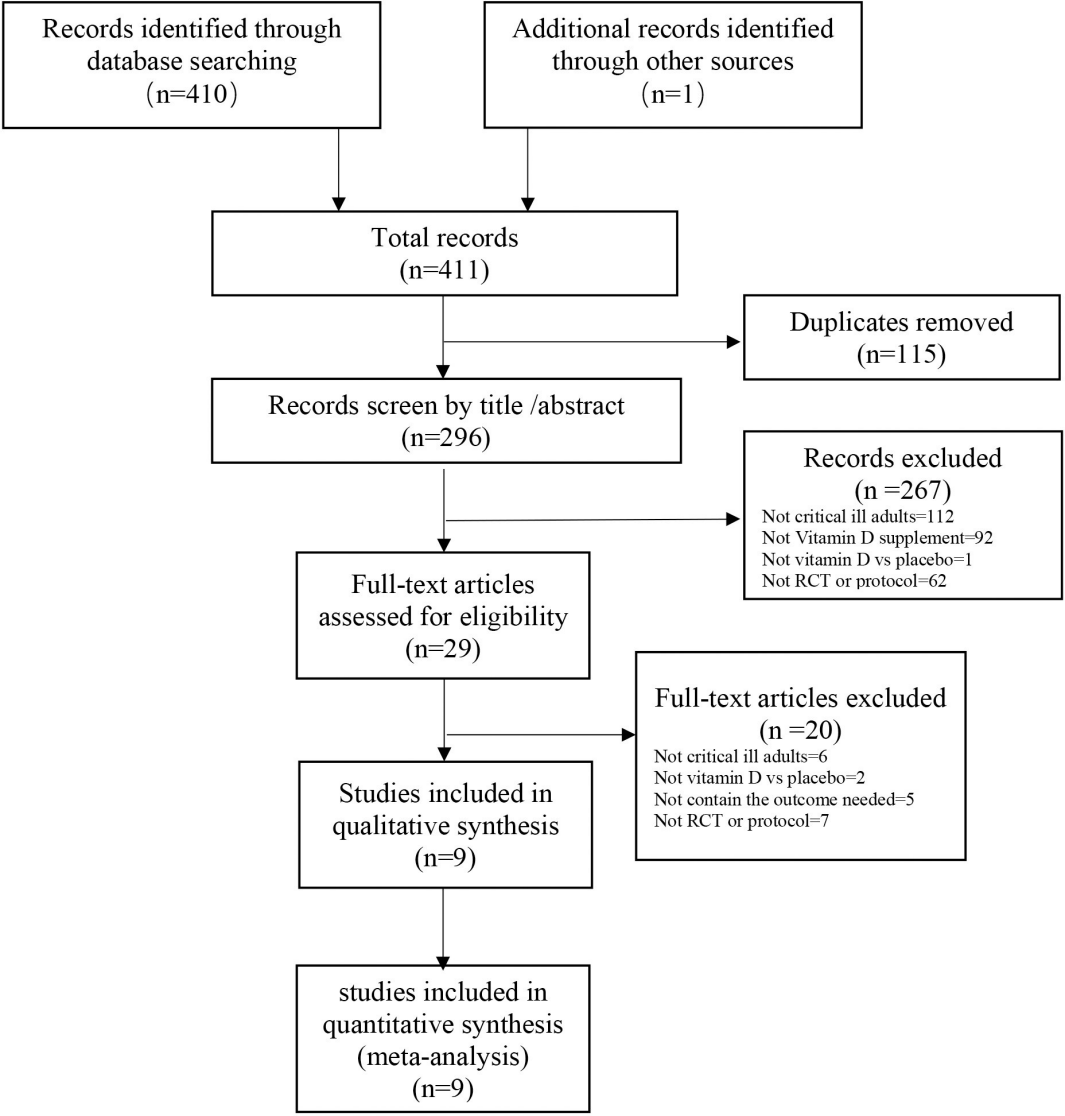
Search strategy and final included and excluded studies.

Our systematic electronic literature search identified a total of 411 reports (Fig 1). After the exclusion of incomplete reports, nine trials [5–8, 10, 17–20] were included in the systematic reviews and meta-analyses.

Details of the trials are summarized in Table 1. All studies were published between 2011 and 2020. The number of recipients ranged from 25 to 1078 patients. The route of administration of vitamin D varied across trials, with six trials [5, 8, 10, 17–19] using oral vitamin D, and one [20] intravenous, two [6, 7] intramuscular. All trials used placebo as a control.

**Table 1:** Characteristics of studies included in the systematic review of vitamin D supplement in critically ill patients.

Trials	Site	patient	BMI mean (SD)	25-hydroxyvitamin D level mean (SD), ng/ml	Patients, N	Mean age, mean (SD), years	Female %	Initial dosages of Vitamin D3(IU)	Longest follow-up for mortality
Amrein 2011	1	Multidisciplinary ICU patients,	29(8.9)	13.6(0.5)	25	62.7(16.3)	24	540000 orally	7 days
Amrein 2014	1	Multidisciplinary ICU patients	27.1(5.3)	13.1(4.1)	475	64.6(14.8)	34	540000 orally	180 days
Ginde 2019	45	Multidisciplinary ICU patients	30.4(10.8)	11.1(4.7)	1078	55.5(16.3)	44	540000 orally	90 days
Han 2016	2	Ventilated ICU patients	NR	21.4(9.1)	31	63.4(17.4)	37	250000 or 500000 orally	84 days
Karsy 2019	1	Neurocritical care patients	NR	14.3(4.4)	274	54(17.2)	43	540000 orally	30 days
Leaf 2014	2	Severe sepsis and septic shock ICU patients	NR	NR	67	63.4(14.1)	45	80 intravenous	28 days
Miri 2019	1	Mechanically ventilated patients	NR	9.7(13.1)	40	53.8(21.9)	27	300000 intramuscular	28 days
Miroliaee 2018	2	Mechanically ventilated patients with pneumonia	NR	18.3(5.5)	51	57.1(19.6)	43	300000 intramuscular	28 days
Quraishi 2015	1	Multidisciplinary ICU patients	28.7(5.9)	17(7.2)	30	63.7(7.6)	40	200000 or 400000 orally	30 days

### Risk of bias and quality of evidence

eFigs 1 and 2 in S1 Appendix present risk-of-bias assessments. eTable 6 in S1 Appendix shows the support for judgment for included trials rated as high or unclear risk of bias. All trials had a low risk of bias [10, 17, 21–27]. Key findings of GRADE assessment of certainty for all outcomes are presented in eTable 4 in S1 Appendix. The quality of evidence of primary outcome was ranked as high.

### Mortality

**Fig 2:**
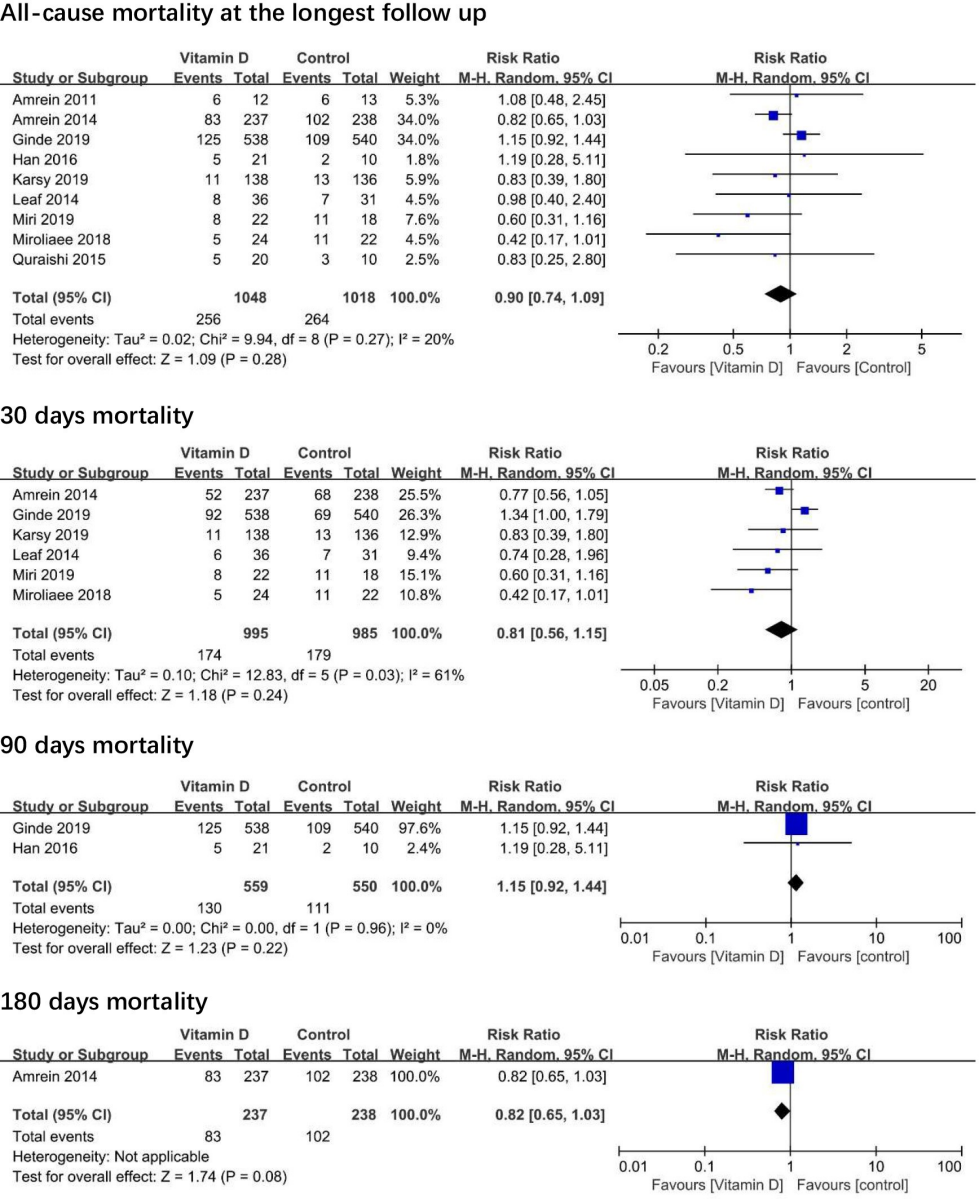
Association of vitamin D versus placebo with mortality.

The association between vitamin D and all-cause mortality is shown in Fig 2. The RR revealed no association between vitamin D and reduced all-cause mortality at the longest follow-up (RR 0.90; 95% CI, 0.74 to 1.09; I^2^ = 20%; 26 fewer events per 1000 [95% CI, -69 to 23]; high-quality evidence).

In trial sequential analyses of all-cause mortality at the longest follow-up, the effect estimated has reached the futility boundary (eFig 3 in S1 Appendix). Sensitivity analyses by excluding trials at each time and using random-effect models yielded similar results (eTable 5 in S1 Appendix). Funnel plot analysis showed no asymmetry (eFig 4 in S1 Appendix), as well as Egger test (p = 0.397) and Begg test (p = 0.602) detected no significant small-study effects.

Subgroup analyses found that there is no significant difference between vitamin D supplementation and placebo in the subgroup analysis of all-cause mortality on dose (≥300000IU or <300000IU) (eFig 5 in S1 Appendix), the baseline 25 hydroxyvitamin D (≥20 and <20 ng/ml) (eFig 6 in S1 Appendix) and the route of administration (oral, intravenous, or intramuscular).

Similarly, the use of vitamin D was not associated with reduced all-cause mortality at 30 days, all-cause mortality at 90 days, all-cause mortality at 180 days, all-cause ICU mortality, or all-cause in-hospital mortality (Fig 2).

### Other outcomes

**Fig 3:**
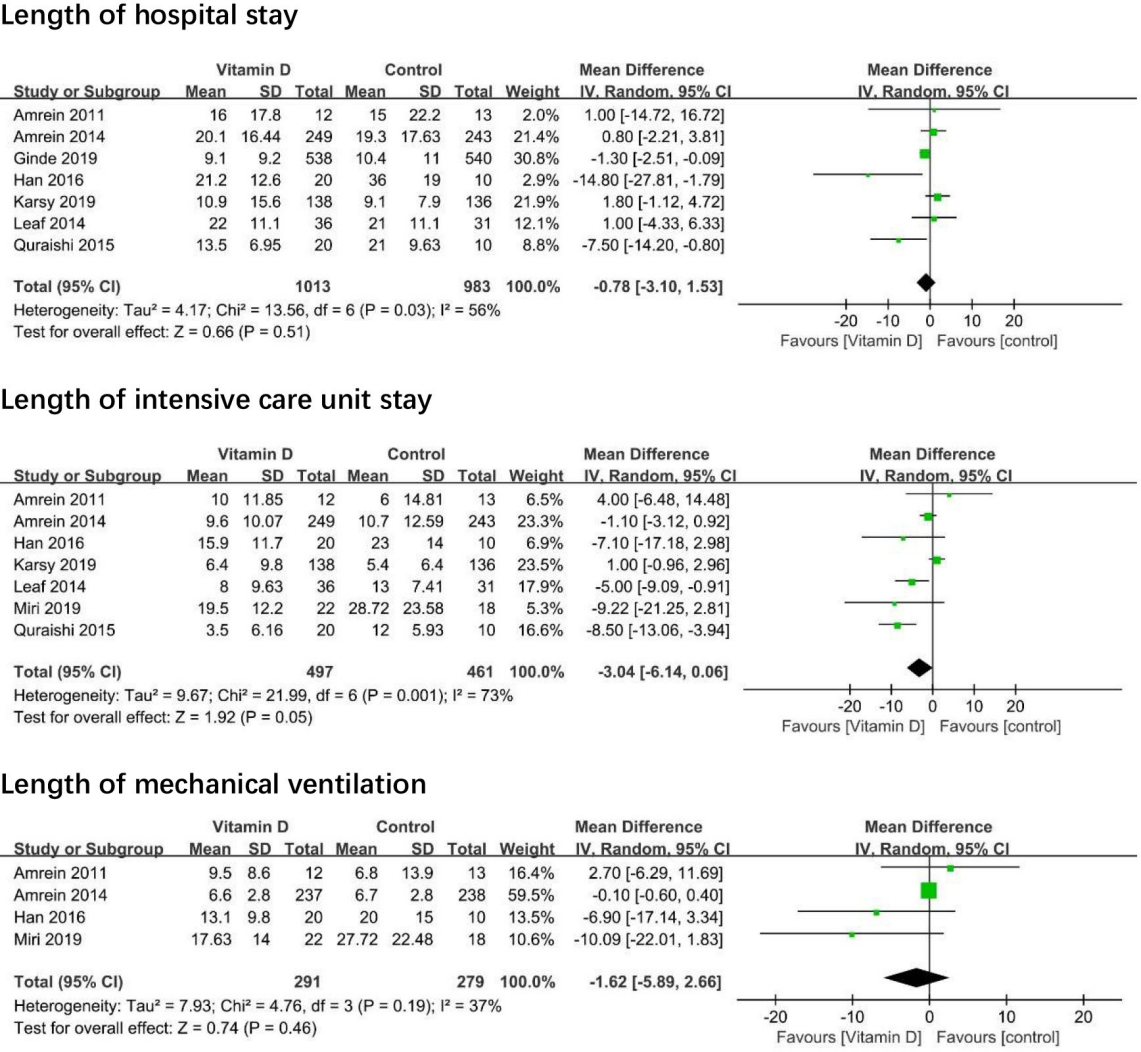
Association of vitamin D versus placebo with length of hospital stay, length of intensive care unit stay, and length of mechanical ventilation.

Seven trials [5, 8, 10, 17–20] reported length of hospital stay, and seven trials [5, 6, 8, 10, 17, 19, 20] reported the length of ICU stay. One thousand thirteen patients take vitamin D and 983 patients did not take vitamin D. The mean difference (MD) between the vitamin D group and placebo group revealed no association between vitamin D and length of hospital stay (MD -0.78; 95% CI -3.10 to 1.53; I^2^ = 56%; moderate-quality evidence; Fig 3) or length of ICU stay (MD -3.04; 95% CI, -6.14 to 0.06; I^2^ = 73%; moderate-quality evidence). Four trials [6, 8, 17, 19] reported the length of mechanical ventilation. Two hundred ninety-one patients take vitamin D and 279 patients not take vitamin D. There was no association between vitamin D and length of mechanical ventilation (MD -1.62; 95%Cl, -5.89 to 2.66; I^2^ = 37%).

## Discussion

### Principal findings

In this meta-analysis of 9 trials with a total of 2066, we did not detect a significant difference between vitamin D and placebo as the treatment of critically ill patients for all-cause mortality. Similarly, vitamin D supplementation does not reduce the length of ICU stay, hospital stay or mechanical ventilation.

### Comparison with other studies

Three systematic reviews and meta-analyses [11–13] have assessed the effect of vitamin D on mortality in critically ill patients in 2017. One review [12] found that vitamin D supplements decreased all-cause mortality at the longest follow-up in analyses of 5 trails [8–10, 17, 20] with a total of 627 patients(odd ratios 0.70, 95% confidence interval 0.50 to 0.98, p = 0.04). The review probably reached more optimistic conclusions as to the use of odd ratios to estimate the effect size. The other two systematic reviews [11, 13] found that vitamin D supplement was trending to decrease all-cause mortality at the longest follow-up in critically ill, but the finding was not statistically significant. The RRs were 0.84 (95%CI 0.66 to 1.06) and 0.77(95%CI 0.58 to 1.03), respectively.

Compared with previous meta-analyses, this study got three times the sample size to make the results more credible. This data improved the precision concerning the treatment effects and provided enough power to the optimum sample size in trial sequential analysis. Moreover, this study has presented absolute as well as relative risks and rank of the quality of evidence of main outcomes. In addition, risk of bias assessment was conducted using the new RoB 2 tool to make the result reliable.

### Limitations

The present study has the following limitations that must be taken into account. First, there were differences across all trials in baseline characteristics (e.g., 25‑hydroxyvitamin D level), the definition of outcomes, and treatment (douse of vitamin D), leading to clinical heterogeneity. Secondly, due to the small number of trials, funnel plots, Egger test, and Begg’s test were non-significant. We cannot rule out the possibility of small-study effects. Thirdly, some trials included patients who personal supplement with vitamin D regularly. For example, in the VIOLET trial [18], 5% of participants in the control group used vitamin D supplementation in the past week. This made it more difficult to distinguish the effect between the treatment and control groups.

## Conclusion

Current evidence shows that vitamin D supplement was not associated with reduced all-cause mortality in critically ill patients. Similarly, vitamin D supplementation does not reduce the length of ICU stay, hospital stay, or mechanical ventilation.

## Supporting information

S1 Appendix (DOCX)
