# Supplementary material for: Association between Vitamin D supplementation and mortality in critically ill patients: A systematic review and meta-analysis of randomized clinical trials
Source: PLoS One. 2020 Dec 14;15(12):e0243768. doi: 10.1371/journal.pone.0243768 (PMC7735584; doi:10.1371/journal.pone.0243768)
Supplement: S1 Appendix — (DOCX) [file pone.0243768.s001.docx]

**Supplemental e-material:**

**eTable 1: PRISMA Checklist.**

**eTable 2: Search strategy.**

**eTable 3: Excluded trials and reason.**

**eTable 4: Summary of Findings and Strength of Evidence (GRADE).**

**eTable 5: Sensitivity analyses.**

**eTable 6: Support for judgement for included trials rated as high or unclear risk of bias.**

**eFigure 1: Risk of bias summary.**

**eFigure 2: Risk of bias graph.**

**eFigure 3: Trial sequential analyses of all-cause mortality at the longest follow up.**

**eFigure 4: Funnel plot analysis of all-cause mortality at the longest follow up.**

**eFigure 5: Subgroup analysis of all-cause mortality at the longest follow-up stratified by baseline 25(OH)D.**

**eFigure 6: Subgroup analysis of all-cause mortality at the longest follow-up stratified by daily dose equivalent.**

**eFigure 7: Subgroup analysis of all-cause mortality at the longest follow-up stratified by route of administration.**

**eFigure 8 Forest plot of hospital mortality and ICU mortality.**

**eTable 1 PRISMA Checklist.**

| **Section/topic** | **#** | **Checklist item** | **Reported on page #** |
| --- | --- | --- | --- |
| **TITLE** | | |  |
| Title | 1 | Identify the report as a systematic review, meta-analysis, or both. | 1 |
| **ABSTRACT** | | |  |
| Structured summary | 2 | Provide a structured summary including, as applicable: background; objectives; data sources; study eligibility criteria, participants, and interventions; study appraisal and synthesis methods; results; limitations; conclusions and implications of key findings; systematic review registration number. | 3 |
| **INTRODUCTION** | | |  |
| Rationale | 3 | Describe the rationale for the review in the context of what is already known. | 4 |
| Objectives | 4 | Provide an explicit statement of questions being addressed with reference to participants, interventions, comparisons, outcomes, and study design (PICOS). | 4 |
| **METHODS** | | |  |
| Protocol and registration | 5 | Indicate if a review protocol exists, if and where it can be accessed (e.g., Web address), and, if available, provide registration information including registration number. | 4 |
| Eligibility criteria | 6 | Specify study characteristics (e.g., PICOS, length of follow-up) and report characteristics (e.g., years considered, language, publication status) used as criteria for eligibility, giving rationale. | 4 |
| Information sources | 7 | Describe all information sources (e.g., databases with dates of coverage, contact with study authors to identify additional studies) in the search and date last searched. | 5 |
| Search | 8 | Present full electronic search strategy for at least one database, including any limits used, such that it could be repeated. | 5 |
| Study selection | 9 | State the process for selecting studies (i.e., screening, eligibility, included in systematic review, and, if applicable, included in the meta-analysis). | 5 |
| Data collection process | 10 | Describe method of data extraction from reports (e.g., piloted forms, independently, in duplicate) and any processes for obtaining and confirming data from investigators. | 5 |
| Data items | 11 | List and define all variables for which data were sought (e.g., PICOS, funding sources) and any assumptions and simplifications made. | 5 |
| Risk of bias in individual studies | 12 | Describe methods used for assessing risk of bias of individual studies (including specification of whether this was done at the study or outcome level), and how this information is to be used in any data synthesis. | 6 |
| Summary measures | 13 | State the principal summary measures (e.g., risk ratio, difference in means). | 5-6 |
| Synthesis of results | 14 | Describe the methods of handling data and combining results of studies, if done, including measures of consistency (e.g., I^2^) for each meta-analysis. | 5-6 |
| Risk of bias across studies | 15 | Specify any assessment of risk of bias that may affect the cumulative evidence (e.g., publication bias, selective reporting within studies). | 5 |
| Additional analyses | 16 | Describe methods of additional analyses (e.g., sensitivity or subgroup analyses, meta-regression), if done, indicating which were pre-specified. | 6-7 |
| **RESULTS** | | | |
| Study selection | 17 | Give numbers of studies screened, assessed for eligibility, and included in the review, with reasons for exclusions at each stage, ideally with a flow diagram. | 7 |
| Study characteristics | 18 | For each study, present characteristics for which data were extracted (e.g., study size, PICOS, follow-up period) and provide the citations. | 7 |
| Risk of bias within studies | 19 | Present data on risk of bias of each study and, if available, any outcome level assessment (see item 12). | 8 |
| Results of individual studies | 20 | For all outcomes considered (benefits or harms), present, for each study: (a) simple summary data for each intervention group (b) effect estimates and confidence intervals, ideally with a forest plot. | 8-9 |
| Synthesis of results | 21 | Present results of each meta-analysis done, including confidence intervals and measures of consistency. | 8-9 |
| Risk of bias across studies | 22 | Present results of any assessment of risk of bias across studies (see Item 15). | 8 |
| Additional analysis | 23 | Give results of additional analyses, if done (e.g., sensitivity or subgroup analyses, meta-regression [see Item 16]). | 8-9 |
| **DISCUSSION** | | | |
| Summary of evidence | 24 | Summarize the main findings including the strength of evidence for each main outcome; consider their relevance to key groups (e.g., healthcare providers, users, and policy makers). | 9-10 |
| Limitations | 25 | Discuss limitations at study and outcome level (e.g., risk of bias), and at review-level (e.g., incomplete retrieval of identified research, reporting bias). | 10-11 |
| Conclusions | 26 | Provide a general interpretation of the results in the context of other evidence, and implications for future research. | 11 |
| **FUNDING** | | | |
| Funding | 27 | Describe sources of funding for the systematic review and other support (e.g., supply of data); role of funders for the systematic review. | 12 |

**etable2 Search strategy.**

| MEDLINE(ovid) | |  |
| --- | --- | --- |
| 1 | exp Vitamin D/ or exp Cholecalciferol/ or exp ergocalciferols/ or exp dihydrotachysterol/ or exp Hydroxycholecalciferols/ | 57922 |
| 2 | (alfacalcidol$ or alphacalcidol$ or colecalciferol$ or cholecalciferol$ or calcifediol$ or calcitriol$ or dihydrotachysterol$ or hydroxyvitamin$ d? or vitamin D?).tw,ot. | 62409 |
| 3 | 1 or 2 | 77037 |
| 4 | exp Critical Illness/ or exp Critical Care/ or exp Intensive Care Units/ or exp Respiration, Artificial/ | 202280 |
| 5 | (ICU or ((intensive or critical) adj3 (care or unit*)) or (critical* adj3 ill*)).mp. or (mechanical* adj3 ventilat*).mp. or (artificial* adj3 respiration*).mp. | 270899 |
| 6 | 4 or 5 | 296968 |
| 7 | exp Randomized Controlled Trials as Topic/ | 133344 |
| 8 | exp Controlled Clinical Trials as Topic/ | 138547 |
| 9 | exp Random Allocation/ | 102064 |
| 10 | exp Double-Blind Method/ | 156019 |
| 11 | exp Single-Blind Method/ | 28065 |
| 12 | Randomized Controlled Trial.pt. | 499478 |
| 13 | Controlled Clinical Trial.pt. | 93503 |
| 14 | 7 or 8 or 9 or 10 or 11 or 12 or 13 | 819826 |
| 15 | exp Animals/ | 22949008 |
| 16 | exp Humans/ | 18280448 |
| 17 | 15 not 16 | 4668560 |
| 18 | (3 and 6 and 14) not 17 | 49 |

| EMBASE(ovid) | |  |
| --- | --- | --- |
| 1 | exp ergocalciferol/ or exp vitamin D/ or exp colecalciferol/ or exp dihydrotachysterol/ or exp 25 hydroxyvitamin D/ or exp hydroxycolecalciferol/ or exp alfacalcidol/ | 137897 |
| 2 | 2. (vitamin* D? or vitamin*D? or cholecalciferol* or colecalciferol* or calcifediol* or calcitriol* or dihydrotachysterol* or hydroxyvitamin* d? or alfacalcidol* or alphacalcidol*).tw,ot. | 105019 |
| 3 | 1 or 2 | 154780 |
| 4 | exp critical illness/ or exp intensive care unit/ or exp intensive care/ or exp artificial ventilation/ | 808501 |
| 5 | (ICU or ((intensive or critical) adj3 (care or unit*)) or (critical* adj3 ill*)).mp. or (mechanical* adj3 ventil*).mp. or (artificial* adj3 respiration*).mp. | 471270 |
| 6 | 4 or 5 | 913719 |
| 7 | exp randomized controlled trial/ | 591966 |
| 8 | exp double blind procedure/ | 169732 |
| 9 | exp single blind procedure/ | 38009 |
| 10 | exp randomization/ | 86159 |
| 11 | exp controlled clinical trial/ | 777140 |
| 12 | 7 or 8 or 9 or 10 or 11 | 878852 |
| 13 | exp animals/ | 25130108 |
| 14 | exp humans/ | 20549702 |
| 15 | 13 not 14 | 4580406 |
| 16 | (3 and 6 and 12) not 15 | 206 |

| Cochrane Central Register of Controlled Trials(ovid) | |  |
| --- | --- | --- |
| 1 | exp Vitamin D/ or exp Cholecalciferol/ or exp Ergocalciferols/ or exp Dihydrotachysterol/ or exp Hydroxycholecalciferols/ | 5139 |
| 2 | (alfacalcidol$ or alphacalcidol$ or colecalciferol$ or cholecalciferol$ or calcifediol$ or calcitriol$ or dihydrotachysterol$ or hydroxyvitamin$ d? or vitamin D?).tw,ot. | 12548 |
| 3 | 1 or 2 | 13240 |
| 4 | exp Critical Illness/ or exp Critical Care/ or exp Intensive Care Units/ or exp Respiration, Artificial/ | 10787 |
| 5 | (ICU or ((intensive or critical) adj3 (care or unit*)) or (critical* adj3 ill*)).mp. or (mechanical* adj3 ventilat*).mp. or (artificial* adj3 respiration*).mp. | 38058 |
| 6 | 4 or 5 | 40106 |
| 7 | 3 and 6 | 155 |

**eTable 3: Excluded trials and reason.**

| **Trials** | **Reason for exclusion** |
| --- | --- |
| Alizadeh et al. 2016^1^ | Not contain the outcome needed |
| Choudhary et al. 2012^2^ | Not critical ill adults |
| Grossmann et al. 2012^3^ | Not critical ill adults |
| Hasanloei et al. 2019^4^ | Not Vitamin D vs Placebo |
| Mann et al. 2014^5^ | Not critical ill adults |
| Smith et al. 2018^6^ | Not contain the outcome needed |
| Han et al. 2017^7^ | Not contain the outcome needed |
| Han et al. 2018^8^ | Not contain the outcome needed |
| Talasaz et al. 2017^9^ | Not critical ill adults |
| Nair et al. 2015^10^ | Not Vitamin D vs Placebo |
| Roh et al. 2006^11^ | Not critical ill adults |
| Jones et al. 2015^12^ | Not contain the outcome needed |
| Amrein et al. 2016^13^ | Not RCT |
| Watkins et al. 2012^14^ | Not RCT |
| Poole et al. 2006^15^ | Not RCT |
| Papaioannou et al. 2007^16^ | Not RCT |
| Amrein et al. 2010^17^ | Not RCT |
| Quraishi et al. 2012^18^ | Not RCT |
| Karin et al. 2016^19^ | Not RCT |
| Parekh et al. 2018^20^ | Not critical ill adults |

**eTable 4. Summary of Findings and Strength of Evidence (GRADE).**

| **Outcome** | **Patients**  **(studies)** | **Relative effect (95% CI)** | **I^2^** | **quality** | **Comments** |
| --- | --- | --- | --- | --- | --- |
| All-cause mortality  at the longest follow-up | 2066  (9) | **0.94**  (0.81 to 1.09) | 20% | high | No |
| 30 days mortality | 1980  (6) | **0.81**  (0.56 to 1.15) | 61% | moderate | Serious inconsistency: evidence of significant interstudy heterogeneity; I^2^=61%, P=0.03) |
| 90 days mortality | 1109  (2) | **1.15**  (0.92 to 1.44) | 0% | high | No |
| 180 days mortality | 475  (1) | **0.82**  (0.65 to 1.03) | NA | high | No |
| Hospital mortality | 573  （3） | **0.81**  (0.63 to 1.05) | 0% | high | No |
| ICU mortality | 542  (2) | **0.87**  (0.65 to 1.18) | 0% | high | No |
| Length of hospital stay | 1996  (7) | MD**-0.78**  (-3.10 to 1.53) | 56% | moderate | Serious inconsistency: evidence of significant interstudy heterogeneity; I^2^=56%, P=0.03) |
| Length of ICU stay | 958  (7) | MD**-3.04**  (-6.14 to 0.06) | 73% | moderate | Serious inconsistency: evidence of significant interstudy heterogeneity; I^2^=73%, P=0.001) |
| Length of mechanical ventilation | 570  (4) | MD**-1.62**  (-5.89 to 2.66) | 37% | moderate | Serious imprecise:95% confidence interval included both important benefit and harm (<2 or >2). |

**eTable 5: Sensitivity analyses of all-cause mortality at the longest follow-up.**

|  | Risk ratio, 95% CI | I^2^ |
| --- | --- | --- |
| Using Fixed-effect models | 0.94 [0.81, 1.09] | 20% |
| Using Absolute risk | -0.03 [-0.09, 0.03] | 33% |
| Excluding trials at each time |  |  |
| Amrein 2011 | 0.88 [0.70, 1.09] | 29% |
| Amrein 2014 | 0.94 [0.73, 1.20] | 12% |
| Ginde 2019 | 0.80 [0.66, 0.96] | 0% |
| Han 2016 | 0.88 [0.71, 1.09] | 29% |
| Karsy 2019 | 0.89 [0.71, 1.11] | 29% |
| Leaf 2014 | 0.88 [0.71, 1.10] | 29% |
| Miri 2019 | 0.93 [0.77, 1.13] | 14% |
| Miroliaee 2018 | 0.95 [0.82, 1.09] | 0% |
| Quraishi 2015 | 0.89 [0.71, 1.10] | 29% |

**eTable 6: Support for judgement for included trials rated as low or unclear risk of bias.**

| Study | Bias | Judgement | Singnaling | Response | Support for judgement |
| --- | --- | --- | --- | --- | --- |
| Amrein 2011 | Randomization process | Low | 1.1 | Y | Sealed envelopes |
|  |  |  | 1.2 | Y |  |
|  |  |  | 1.3 | N | There is no significant difference between groups |
|  | Deviations from intended interventions | Low | 2.1 | N | The study medication was prepared, labelled and randomized by a pharmacist and physician not involved in the trial. |
|  |  |  | 2.2 | N |  |
|  |  |  | 2.6 | Y | ITT analysis |
|  | Missing outcome data | Low | 3.1 | Y | No loss to follow-up |
|  | Measurement of the outcome | Low | 4.1 | N | Objective outcome |
|  |  |  | 4.2 | N | Objective outcome |
|  |  |  | 4.3 | N | Objective outcome |
|  | Selection of the reported result | Low | 5.1 | PY | We can infer from the rigorous style, though no information about the question |
|  |  |  | 5.2 | N | This outcome need not to be measured in multiple ways |
|  |  |  | 5.3 | N | This outcome need not to be analysed in multiple ways |
|  | Overall Bias | Low | NA | | |
| Amrein 2014 | Randomization process | Low | 1.1 | Y | Computerised random number by using generator program |
|  |  |  | 1.2 | Y |  |
|  |  |  | 1.3 | N | there is no significant difference between groups |
|  | Deviations from intended interventions | Low | 2.1 | N | The study medication was prepared, labelled and randomized by a pharmacist and physician not involved in the trial. |
|  |  |  | 2.2 | N |  |
|  |  |  | 2.6 | Y | ITT analysis |
|  | Missing outcome data | Low | 3.1 | Y | Participants with Incomplete outcome data less than 20% |
|  | Measurement of the outcome | Low | 4.1 | N | Objective outcome |
|  |  |  | 4.2 | N | Objective outcome |
|  |  |  | 4.3 | N | Objective outcome |
|  | Selection of the reported result | Low | 5.1 | Y | The planned outcome is consistent with the reported outcome |
|  |  |  | 5.2 | N | This outcome need not to be measured in multiple ways |
|  |  |  | 5.3 | N | This outcome need not to be analysed in multiple ways |
|  | Overall Bias | Low | NA | | |
| Ginde 2019 | Randomization process | Low | 1.1 | Y | Computerised random number by using generator program；central electronic system。 |
|  |  |  | 1.2 | Y |  |
|  |  |  | 1.3 | N | There is no significant difference between groups |
|  | Deviations from intended interventions | Low | 2.1 | N | The study medication was prepared, labelled and randomized by a pharmacist and physician not involved in the trial. |
|  |  |  | 2.2 | N |  |
|  |  |  | 2.6 | Y | ITT analysis |
|  | Missing outcome data | Low | 3.1 | Y | Participants with Incomplete outcome data less than 20% |
|  | Measurement of the outcome | Low | 4.1 | N | Objective outcome |
|  |  |  | 4.2 | N | Objective outcome |
|  |  |  | 4.3 | N | Objective outcome |
|  | Selection of the reported result | Low | 5.1 | Y | The planned outcome is consistent with the reported outcome |
|  |  |  | 5.2 | N | This outcome need not to be measured in multiple ways |
|  |  |  | 5.3 | N | This outcome need not to be analysed in multiple ways |
|  | Overall Bias | Low | NA | | |
| Han 2016 | Randomization process | Low | 1.1 | Y | a blinded block randomization schedule |
|  |  |  | 1.2 | PY |  |
|  |  |  | 1.3 | N | There is no significant difference between groups |
|  | Deviations from intended interventions | Low | 2.1 | N | blinded to the patients and investigators |
|  |  |  | 2.2 | N |  |
|  |  |  | 2.6 | Y | ITT analysis |
|  | Missing outcome data | Low | 3.1 | Y | Participants with Incomplete outcome data less than 20% |
|  | Measurement of the outcome | Low | 4.1 | N | Objective outcome |
|  |  |  | 4.2 | N | Objective outcome |
|  |  |  | 4.3 | N | Objective outcome |
|  | Selection of the reported result | Low | 5.1 | Y | The planned outcome is consistent with the reported outcome |
|  |  |  | 5.2 | N | This outcome need not to be measured in multiple ways |
|  |  |  | 5.3 | N | This outcome need not to be analysed in multiple ways |
|  | Overall Bias | Low | NA | | |
| Karsy 2019 | Randomization process | Low | 1.1 | Y | Random number generator,managed by the investigational drug pharmacy. |
|  |  |  | 1.2 | Y |  |
|  |  |  | 1.3 | N | There is no significant difference between groups |
|  | Deviations from intended interventions | Low | 2.1 | N | blinded to the patients and investigators |
|  |  |  | 2.2 | N |  |
|  |  |  | 2.6 | Y | ITT analysis |
|  | Missing outcome data | Low | 3.1 | Y | Participants with Incomplete outcome data less than 20% |
|  | Measurement of the outcome | Low | 4.1 | N | Objective outcome |
|  |  |  | 4.2 | N | Objective outcome |
|  |  |  | 4.3 | N | Objective outcome |
|  | Selection of the reported result | Low | 5.1 | Y | The planned outcome is consistent with the reported outcome |
|  |  |  | 5.2 | N | This outcome need not to be measured in multiple ways |
|  |  |  | 5.3 | N | This outcome need not to be analysed in multiple ways |
|  | Overall Bias | Low | NA | | |
| Leaf 2014 | Randomization process | Low | 1.1 | Y | Computerised random number by using generator program,Central concealment. |
|  |  |  | 1.2 | Y |  |
|  |  |  | 1.3 | N | There is no significant difference between groups |
|  | Deviations from intended interventions | Low | 2.1 | PN | Nurses and physicians were blinded to study groups |
|  |  |  | 2.2 | N |  |
|  |  |  | 2.6 | Y | ITT analysis |
|  | Missing outcome data | Low | 3.1 | Y | No loss to follow-up |
|  | Measurement of the outcome | Low | 4.1 | N | Objective outcome |
|  |  |  | 4.2 | N | Objective outcome |
|  |  |  | 4.3 | N | Objective outcome |
|  | Selection of the reported result | Low | 5.1 | Y | The planned outcome is consistent with the reported outcome |
|  |  |  | 5.2 | N | This outcome need not to be measured in multiple ways |
|  |  |  | 5.3 | N | This outcome need not to be analysed in multiple ways |
|  | Overall Bias | Low | NA | | |
| Miri 2019 | Randomization process | Low | 1.1 | Y | Permuted block |
|  |  |  | 1.2 | PY |  |
|  |  |  | 1.3 | N | There is no significant difference between groups |
|  | Deviations from intended interventions | Low | 2.1 | N | Nurses and physicians were blinded to study groups |
|  |  |  | 2.2 | N |  |
|  |  |  | 2.6 | Y | ITT analysis |
|  | Missing outcome data | Low | 3.1 | Y | Participants with Incomplete outcome data less than 20% |
|  | Measurement of the outcome | Low | 4.1 | N | Objective outcome |
|  |  |  | 4.2 | N | Objective outcome |
|  |  |  | 4.3 | N | Objective outcome |
|  | Selection of the reported result | Low | 5.1 | PY | We can infer from the rigorous style, though no information about the question |
|  |  |  | 5.2 | N | This outcome need not to be measured in multiple ways |
|  |  |  | 5.3 | N | This outcome need not to be analysed in multiple ways |
|  | Overall Bias | Low | NA | | |
| Miroliaee 2018 | Randomization process | Low | 1.1 | PY | No information regarding the method,but we can judge by context. |
|  |  |  | 1.2 | PY |  |
|  |  |  | 1.3 | N | There is no significant difference between groups |
|  | Deviations from intended interventions | Low | 2.1 | PN | No information regarding the method,but we can judge by context. |
|  |  |  | 2.2 | PN |  |
|  |  |  | 2.6 | Y | ITT analysis |
|  | Missing outcome data | Low | 3.1 | Y | Participants with Incomplete outcome data less than 20% |
|  | Measurement of the outcome | Low | 4.1 | N | Objective outcome |
|  |  |  | 4.2 | N | Objective outcome |
|  |  |  | 4.3 | N | Objective outcome |
|  | Selection of the reported result | Low | 5.1 | PY | We can infer from the rigorous style, though no information about the question |
|  |  |  | 5.2 | N | This outcome need not to be measured in multiple ways |
|  |  |  | 5.3 | N | This outcome need not to be analysed in multiple ways |
|  | Overall Bias | Low | NA | | |
| Quraishi 2015 | Randomization process | Low | 1.1 | Y | Computerised random number by using generator program,  computer dispense the coded syringes directly. |
|  |  |  | 1.2 | Y |  |
|  |  |  | 1.3 | N | There is no significant difference between groups |
|  | Deviations from intended interventions | Low | 2.1 | N | The study medication was prepared, labelled and randomized by a pharmacist and physician not involved in the trial. |
|  |  |  | 2.2 | N |  |
|  |  |  | 2.6 | Y | ITT analysis |
|  | Missing outcome data | Low | 3.1 | Y | No loss to follow-up |
|  | Measurement of the outcome | Low | 4.1 | N | Objective outcome |
|  |  |  | 4.2 | N | Objective outcome |
|  |  |  | 4.3 | N | Objective outcome |
|  | Selection of the reported result | Low | 5.1 | Y | The planned outcome is consistent with the reported outcome |
|  |  |  | 5.2 | N | This outcome need not to be measured in multiple ways |
|  |  |  | 5.3 | N | This outcome need not to be analysed in multiple ways |
|  | Overall Bias | Low | NA | | |

**eFigure 1: Risk of bias summary.**


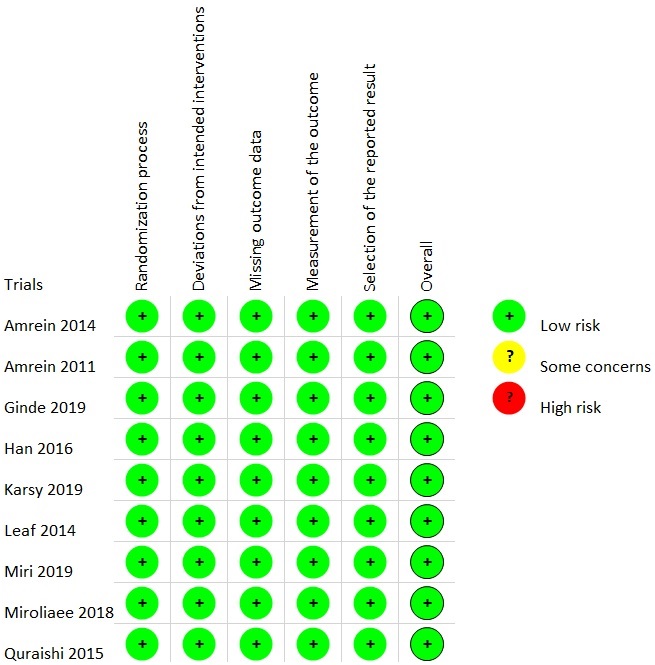


**eFigure 2: Risk of bias graph.**


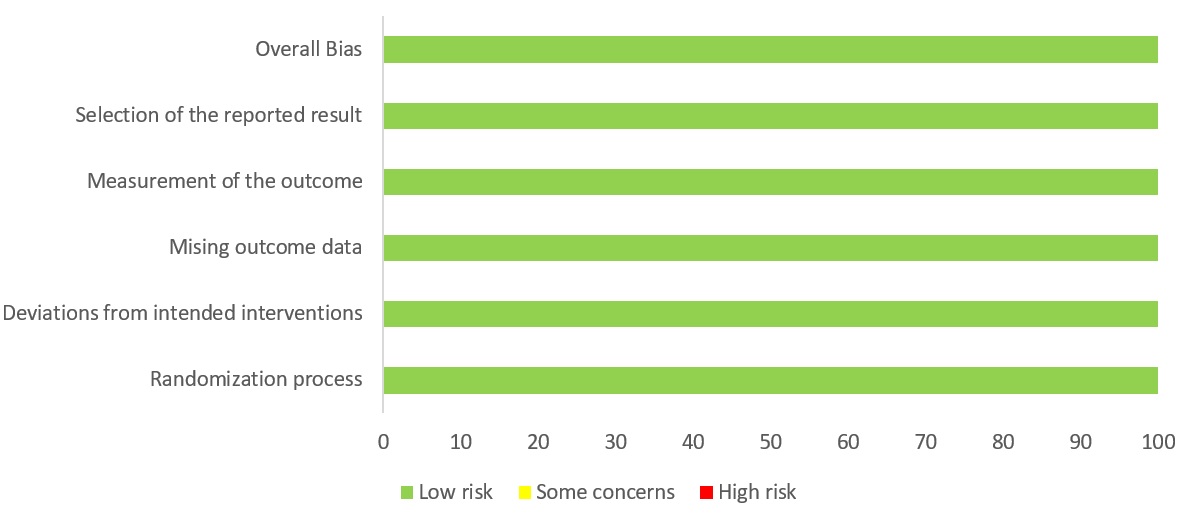


**eFigure 3 Trial sequential analyses of all-cause mortality at the longest follow-up.**


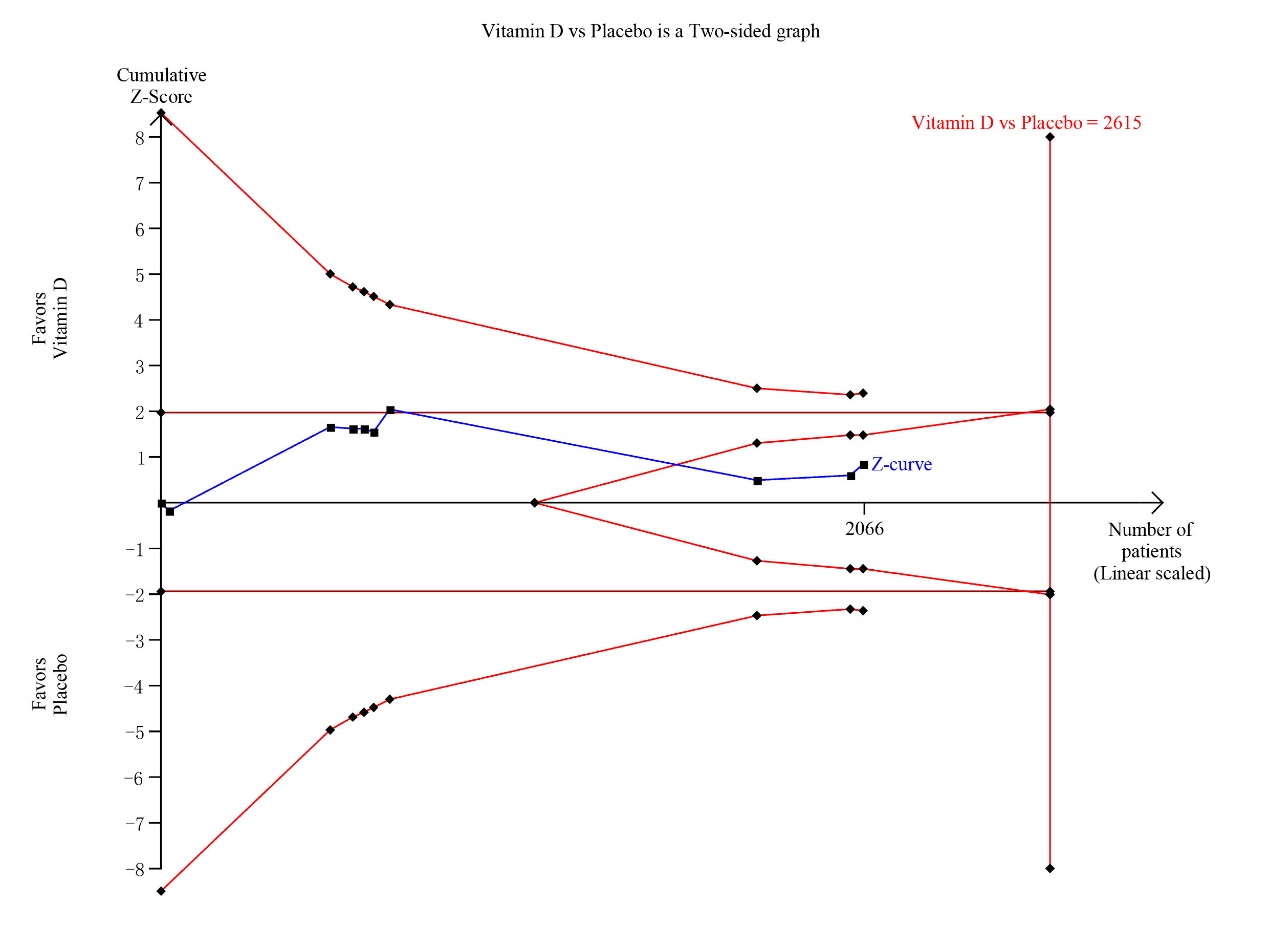


**eFigure 4 Funnel plot analysis of all-cause mortality at the longest follow-up.**


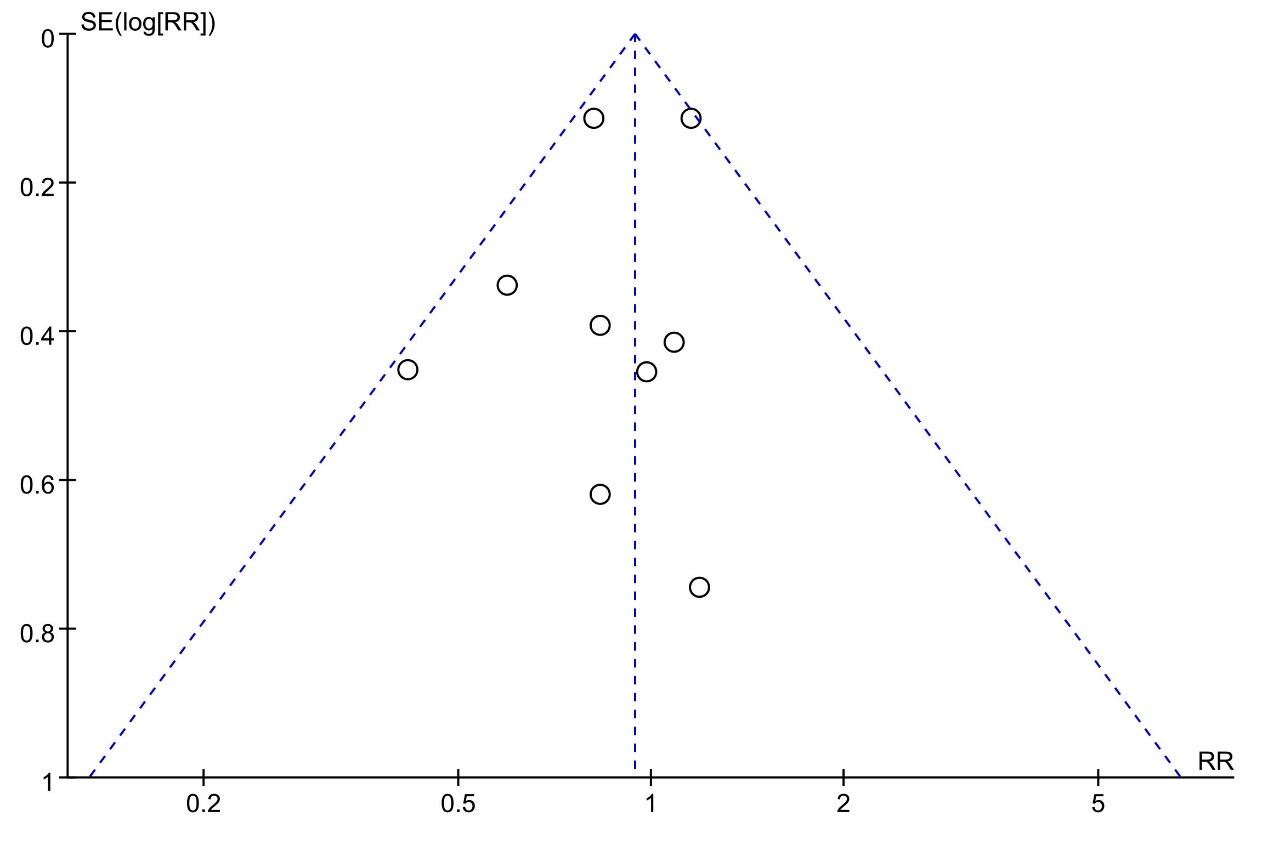


**eFigure 5: Subgroup analysis of all-cause mortality at the longest follow-up stratified by baseline 25(OH)D.**


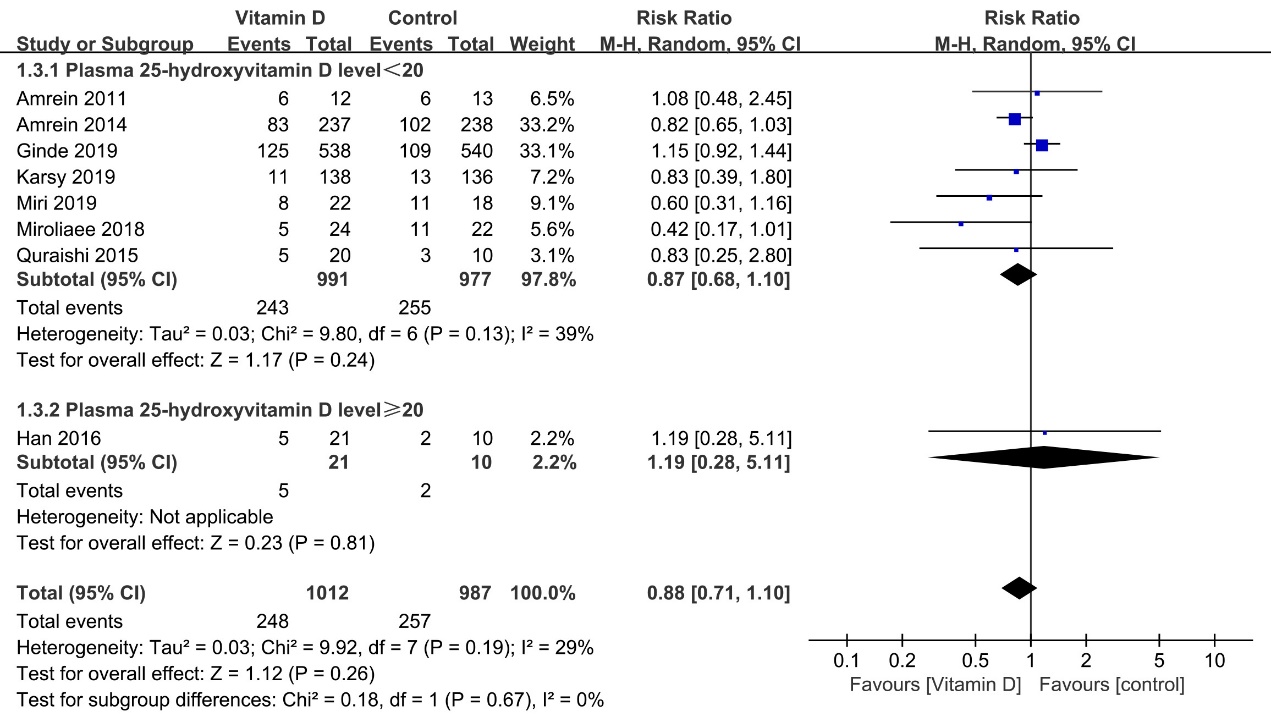


**eFigure 6: Subgroup analysis of all-cause mortality at the longest follow-up stratified by daily dose equivalent.**

**
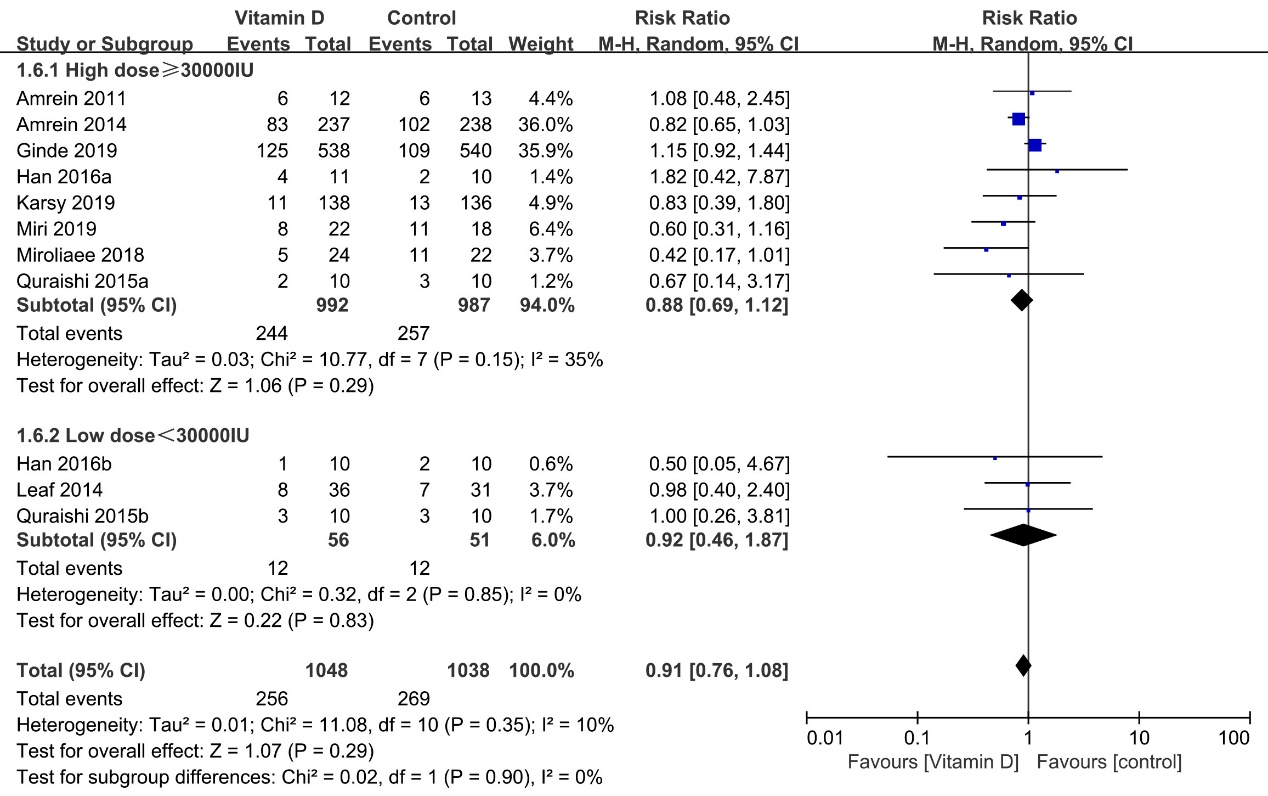
**

**eFigure 7: Subgroup analysis of all-cause mortality at the longest follow-up stratified by route of administration.**


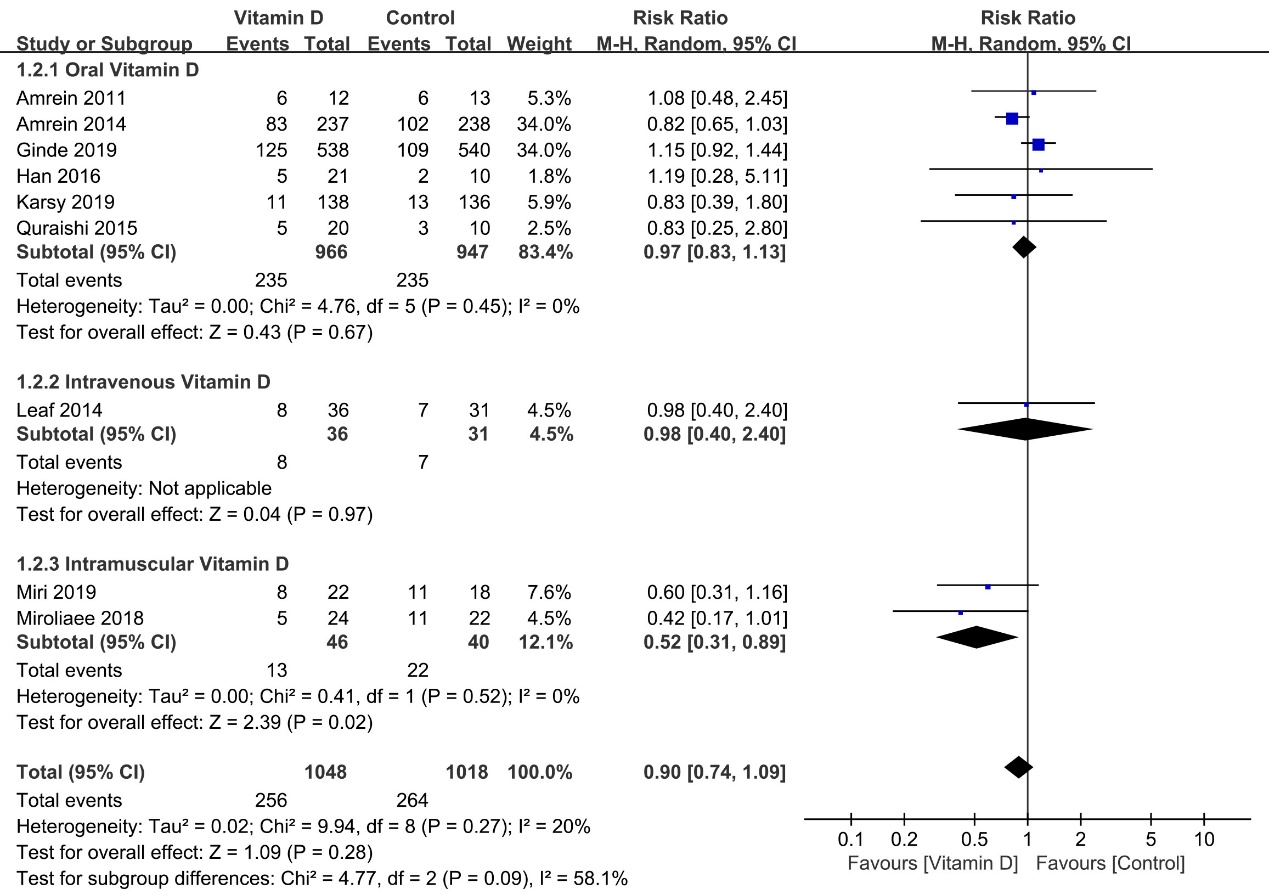


**eFigure 7 Forest plot of** **hospital mortality and ICU mortality.**


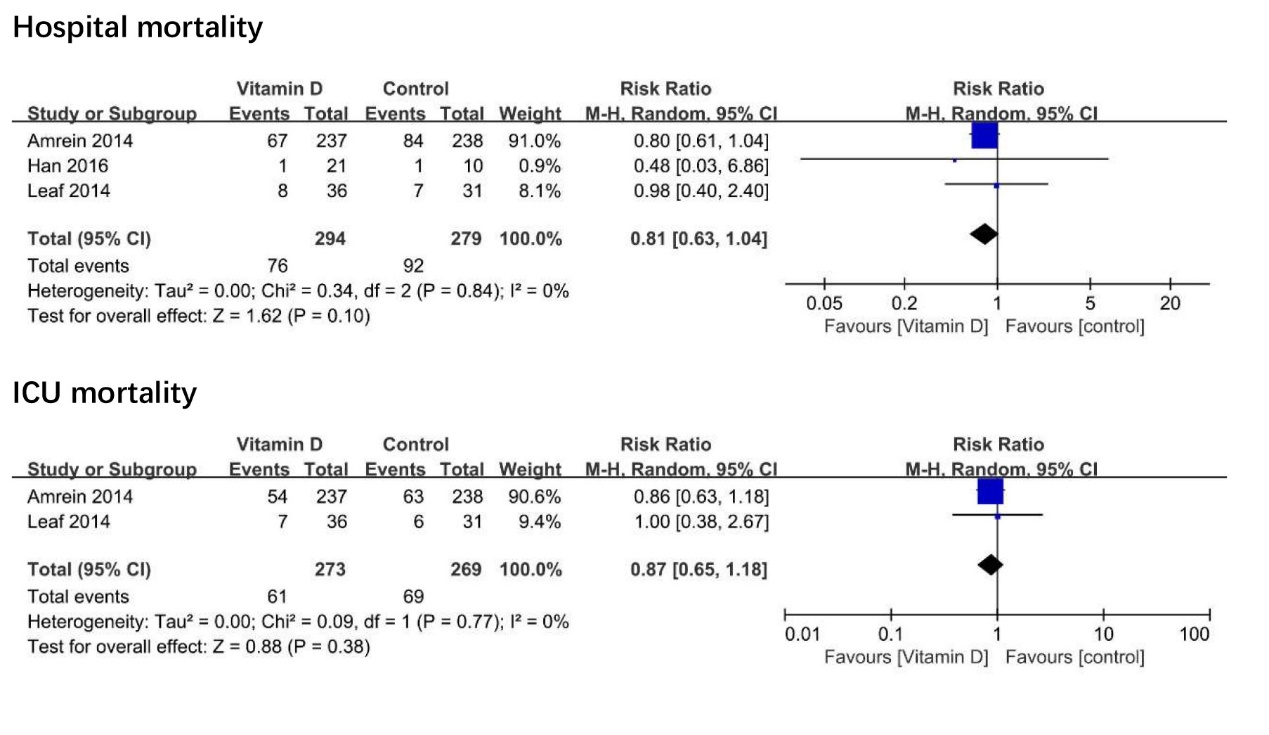


1. Alizadeh N, Khalili H, Mohammadi M, Abdollahi A, Ala S. Effect of vitamin D on stress-induced hyperglycaemia and insulin resistance in critically ill patients. International journal of clinical practice 2016;70:396-405.

2. Choudhary N, Gupta P. Vitamin D supplementation for severe Pneumonia - A randomized controlled trial. Indian Pediatrics 2012;49:449-454.

3. Grossmann RE, Zughaier S, Kumari M, et al. Clinical responses to a novel vitamin D supplementation strategy in adult CF patients hospitalized for pulmonary exacerbation. Pediatric Pulmonology 2011;46:404.

4. Hasanloei MAV, Rahimlou M, Eivazloo A, Sane S, Ayremlou P, Hashemi R. Effect of Oral Versus Intramuscular Vitamin D Replacement on Oxidative Stress and Outcomes in Traumatic Mechanical Ventilated Patients Admitted to Intensive Care Unit. Nutrition in Clinical Practice 2019.

5. Mann MC, Exner DV, Hemmelgarn BR, et al. The VITAH Trial Vitamin D supplementation and cardiac autonomic tone in hemodialysis: A blinded, randomized controlled trial. BMC Nephrology 2014;15:129.

6. Smith EM, Jones JL, Han JE, et al. High-Dose Vitamin D3 Administration Is Associated With Increases in Hemoglobin Concentrations in Mechanically Ventilated Critically Ill Adults: A Pilot Double-Blind, Randomized, Placebo-Controlled Trial. Journal of Parenteral and Enteral Nutrition 2018;42:87-94.

7. Han JE, Alvarez JA, Jones JL, et al. Impact of high-dose vitamin D3 on plasma free 25-hydroxyvitamin D concentrations and antimicrobial peptides in critically ill mechanically ventilated adults. Nutrition (Burbank, Los Angeles County, Calif) 2017;38:102-108.

8. Han JE, Alvarez JA, Staitieh B, et al. Oxidative stress in critically ill ventilated adults: effects of vitamin D3 and associations with alveolar macrophage function. European journal of clinical nutrition 2018;72:744-751.

9. Talasaz AH, Daei MD, Karimi AK. Potential role of vitamin D supplementation in the prevention of atrial fibrillation after coronary artery bypass grafting in vitamin D deficient patients. European Heart Journal 2017;38:579-580.

10. Nair P, Venkatesh B, Lee P, et al. A Randomized Study of a Single Dose of Intramuscular Cholecalciferol in Critically Ill Adults. Critical care medicine 2015;43:2313-2320.

11. Roh JL, Park CI. Routine oral calcium and vitamin D supplements for prevention of hypocalcemia after total thyroidectomy. American Journal of Surgery 2006;192:675-678.

12. Jones J AJHJHLCEBLTVMGZT. Effect of high-dose vitamin D on antimicrobial peptides in ventilator-dependent critically ill patients. FASEB journal 2015;29.

13. Amrein K, McNally JD, Dobnig H, Pieber TR. High-dose cholecalciferol in critically ill patients with liver cirrhosis. Journal of internal medicine 2016;279:309-310.

14. Watkins RR. Investigating the association between vitamin D deficiency and sepsis: Challenges and future prospects. Expert Review of Anti-Infective Therapy 2012;10:723-725.

15. Poole KES, Compston JE. Osteoporosis and its management. British Medical Journal 2006;333:1251-1256.

16. Papaioannou A, Kennedy CC, Dolovich L, Lau E, Adachi JD. Patient adherence to osteoporosis medications: Problems, consequences and management strategies. Drugs and Aging 2007;24:37-55.

17. Amrein K, Amrein S, Holl A, et al. Serum 25-hydroxyvitamin d status of critically ill patients is predictive of hospital mortality independent of saps ii. Intensive Care Medicine 2010;36:S331.

18. Quraishi SA, Camargo CA, Jr. Vitamin D in acute stress and critical illness. Current opinion in clinical nutrition and metabolic care 2012;15:625-634.

19. Amrein K, Parekh D, Westphal S, et al. Effect of high-dose vitamin D on 28-day mortality in adult critically ill patients with severe vitamin D deficiency. Effect of high-dose vitamin D3 on 28-day mortality in adult critically ill patients with severe vitamin D deficiency: a multicenter, placebo-controlled double-blind phase III RCT - VITDALIZE 2016.

20. Parekh D, Dancer RCA, Scott A, et al. Vitamin D to Prevent Lung Injury Following Esophagectomy-A Randomized, Placebo-Controlled Trial. Critical care medicine 2018;46:e1128-e1135.
